# Neoatherosclerosis prediction using plaque markers in intravascular optical coherence tomography images

**DOI:** 10.3389/fcvm.2022.1079046

**Published:** 2022-12-14

**Authors:** Juhwan Lee, Gabriel T. R. Pereira, Issam Motairek, Justin N. Kim, Vladislav N. Zimin, Luis A. P. Dallan, Ammar Hoori, Sadeer Al-Kindi, Giulio Guagliumi, David L. Wilson

**Affiliations:** ^1^Department of Biomedical Engineering, Case Western Reserve University, Cleveland, OH, United States; ^2^Cardiovascular Imaging Core Laboratory, Harrington Heart and Vascular Institute, University Hospitals Cleveland Medical Center, Cleveland, OH, United States; ^3^Cardiovascular Department, Galeazzi San’Ambrogio Hospital, Innovation District, Milan, Italy; ^4^Department of Radiology, Case Western Reserve University, Cleveland, OH, United States

**Keywords:** intravascular optical coherence tomography, neoatherosclerosis, plaque characteristics, OCTOPUS, fibrous cap surface area

## Abstract

**Introduction:**

In-stent neoatherosclerosis has emerged as a crucial factor in post-stent complications including late in-stent restenosis and very late stent thrombosis. In this study, we investigated the ability of quantitative plaque characteristics from intravascular optical coherence tomography (IVOCT) images taken just prior to stent implantation to predict neoatherosclerosis after implantation.

**Methods:**

This was a sub-study of the TRiple Assessment of Neointima Stent FOrmation to Reabsorbable polyMer with Optical Coherence Tomography (TRANSFORM-OCT) trial. Images were obtained before and 18 months after stent implantation. Final analysis included images of 180 lesions from 90 patients; each patient had images of two lesions in different coronary arteries. A total of 17 IVOCT plaque features, including lesion length, lumen (e.g., area and diameter); calcium (e.g., angle and thickness); and fibrous cap (FC) features (e.g., thickness, surface area, and burden), were automatically extracted from the baseline IVOCT images before stenting using dedicated software developed by our group (OCTOPUS). The predictive value of baseline IVOCT plaque features for neoatherosclerosis development after stent implantation was assessed using univariate/multivariate logistic regression and receiver operating characteristic (ROC) analyses.

**Results:**

Follow-up IVOCT identified stents with (*n* = 19) and without (*n* = 161) neoatherosclerosis. Greater lesion length and maximum calcium angle and features related to FC were associated with a higher prevalence of neoatherosclerosis after stent implantation (*p* < 0.05). Hierarchical clustering identified six clusters with the best prediction *p*-values. In univariate logistic regression analysis, maximum calcium angle, minimum calcium thickness, maximum FC angle, maximum FC area, FC surface area, and FC burden were significant predictors of neoatherosclerosis. Lesion length and features related to the lumen were not significantly different between the two groups. In multivariate logistic regression analysis, only larger FC surface area was strongly associated with neoatherosclerosis (odds ratio 1.38, 95% confidence interval [CI] 1.05–1.80, *p* < 0.05). The area under the ROC curve was 0.901 (95% CI 0.859–0.946, *p* < 0.05) for FC surface area.

**Conclusion:**

Post-stent neoatherosclerosis can be predicted by quantitative IVOCT imaging of plaque characteristics prior to stent implantation. Our findings highlight the additional clinical benefits of utilizing IVOCT imaging in the catheterization laboratory to inform treatment decision-making and improve outcomes.

## 1 Introduction

Percutaneous coronary intervention (PCI) is the most common revascularization procedure for treating coronary artery disease, with an average of 660,000 procedures performed per year (1). Despite the advent of newer-generation drug-eluting stents that markedly reduced late thrombotic events (2, 3), PCI continues to be associated with late stent failure and stent thrombosis or restenosis (4–7). *De novo* development of atherosclerosis within the stent neointimal region, i.e., in-stent neoatherosclerosis (8–10), has emerged as a crucial contributing factor to late vascular complications including late in-stent restenosis and very late stent thrombosis (4–7, 10–16).

Neoatherosclerosis is characterized histologically by the accumulation of lipid-laden foamy macrophages with or without necrotic core formation and/or calcification within the neointimal tissue (17). Multiple studies have analyzed the characteristics and formation of neoatherosclerosis using intravascular imaging modalities, such as intravascular ultrasound (IVUS), near-infrared spectroscopy, and intravascular optical coherence tomography (IVOCT). IVUS and IVOCT can improve procedural success, and these techniques are recommended by current guidelines to evaluate the mechanisms of stent failure (18, 19). However, findings from IVUS should be interpreted with caution due to insufficient resolution (150–200 μm) to enable reliable tissue characterization. IVOCT, which has higher resolution (10–20 μm), has become one of the best methods to assess the neointimal tissue (20–22). IVOCT allows for comprehensive assessment of morphological characteristics of neoatherosclerotic plaque composition, such as macrophage infiltration, in-stent calcification, and neointimal rupture (21). On IVOCT images, neoatherosclerosis is mainly limited to lipidic and calcific neointima (23, 24): lipidic neointima appears as a signal-poor region with diffused borders and a fast IVOCT signal drop-off, whereas calcific neointima appears as a signal-poor region with sharply delineated borders (25, 26).

Several studies have identified key factors associated with neoatherosclerosis risk after PCI, including stent type (15, 17, 23, 27–32), clinical factors (24, 27, 33), plaque characteristics (11, 34–36), and stent characteristics (37); however, the mechanism underlying accelerated development of neoatherosclerosis after stent implantation remains unknown. Given that IVOCT provides better resolution of the neointima and neoatherosclerotic plaque characteristics than any other imaging modality, we investigated the value of quantitative IVOCT measurements before stent implantation for predicting post-stent neoatherosclerosis. Building on our previous work in the analysis of atherosclerotic plaque (38–51), we used a number of computational tools (i.e., machine learning, deep learning, and statistical modeling) to evaluate serial IVOCT images collected as part of a large clinical study. Using a dedicated software (OCTOPUS) (38), we performed plaque characterization in the baseline IVOCT images, computed IVOCT plaque features, and determined their association with the development of neoatherosclerosis after stent implantation.

## 2 Materials and methods

### 2.1 Study population

This was a sub-study of the TRiple Assessment of Neointima Stent FOrmation to Reabsorbable polyMer with Optical Coherence Tomography (TRANSFORM-OCT) trial (52), a prospective, randomized, open-label, assessor-blinded, controlled study of patients with multivessel disease undergoing staged PCI with stent implantation at two hospitals in Italy. Patients with stable angina and documented ischemia or acute coronary syndrome who had undergone IVOCT examination were eligible for the study. Major exclusion criteria were the presence of unprotected left main disease, chronic total occlusion, baseline serum creatinine >2.0 mg/dL, life expectancy <18 months, and unsuitable for OCT imaging (at the investigator’s discretion). The full list of inclusion and exclusion criteria is provided in elsewhere (52). Final analysis included 180 lesions among 90 patients. Each patient had a staged PCI with the culprit lesion treated at the index timepoint and a second lesion treated 3 months later. This study was conducted in accordance with the Declaration of Helsinki and with approval from the Institutional Review Board of University Hospitals Cleveland Medical Center (Cleveland, OH, USA). Written informed consent was obtained from all patients.

### 2.2 IVOCT imaging

**FIGURE 1:**
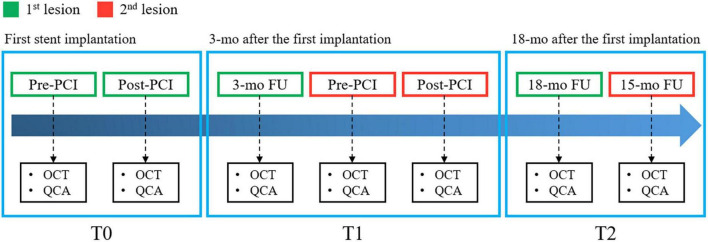
Diagram of intravascular optical coherence tomography (IVOCT) imaging timing, performed before and after first stent implantation (T0), 3 months later before and after second stent implantation (T1), and 18 months after the first implantation (T2).

**FIGURE 2:**
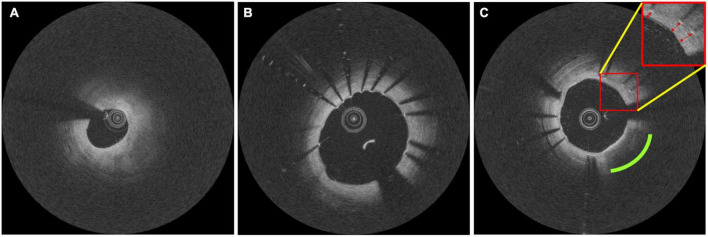
Representative intravascular optical coherence tomography (IVOCT) images documenting the formation of in-stent neoatherosclerosis in one study participant. Images taken at **(A)** baseline, **(B)** 3-month follow-up, and **(C)** 18-month follow-up. Follow-up pullbacks **(B,C)** were manually registered to the baseline pullback **(A)**. In panel **(C)**, the green and red markers indicate the development of lipidic neoatherosclerosis and the formation of neointima, respectively.

Intravascular optical coherence tomography images were acquired with a frequency-domain OCT system (ILUMIEN OPTIS, Abbott Vascular, Santa Clara, CA, USA), which has a tunable laser light source sweeping from 1,250 to 1,360 nm at a frame rate of 180 fps. Baseline and follow-up IVOCT pullbacks were obtained for two independent lesions per patient. The first lesion was treated immediately after randomization, and the entire lesion was stented with one or more allocated stents. Similarly, the second lesion in a different coronary artery was covered by implanting the same allocated stent 3 months from randomization (Figure 1) (52). IVOCT imaging of the first lesion was obtained before and immediately after initial stent implantation (T0) and at 18 months after initial stent implantation (T2). The second lesion was imaged 3 months after randomization, before and after the second stent implantation (T1) and at 15 months after the second stent implantation (T2). Details of IVOCT image acquisition are provided elsewhere (52). Figure 2 shows a representative example of pre- and post-stent IVOCT images in a single study participant with evidence of neoatherosclerosis.

### 2.3 Plaque feature extraction from pre-stent IVOCT images

Atherosclerotic plaque characteristics in pre-stent images were analyzed using a dedicated software (Optical Coherence TOmography PlaqUe and Stent, OCTOPUS), developed by our group (38, 41, 43–46). A total of 17 IVOCT features, including lesion length and several lumen, calcium, and fibrous cap (FC) features, were extracted from the baseline IVOCT images (before stent implantation, T0 and T1). All features were automatically computed using OCTOPUS. Lesion length was defined as the length of vessel segment where the stent was implanted. Lumen features included minimum/mean lumen area and lumen diameter; calcium features consisted of maximum/minimum calcium angle, calcium thickness, and calcium depth; and FC features comprised maximum/minimum FC angle, minimum FC thickness, maximum FC area, FC surface area, and FC burden. FC angle, FC thickness, and FC area were calculated from each IVOCT frame, whereas FC surface area and FC burden were calculated from the entire lesion. FC thickness was automatically calculated by identifying the luminal and abluminal boundaries based on the method developed by our group (42, 48). Briefly, the lumen boundary was obtained using the deep learning segmentation method (41). We used dynamic programming (42) to segment the abluminal boundary since it has a gradual transition of pixel intensity from bright to dark. FC surface area was defined as the amount of FC area covering the surface of the lumen. FC burden was computed as FC area divided by the surface area of the lumen. Table 1 shows the 17 IVOCT features analyzed as predictors of post-stent neoatherosclerosis.

**TABLE 1:** Intravascular optical coherence tomography (IVOCT) plaque features measured as potential predictors of post-stent neoatherosclerosis.

N	Features	
1	Lesion length (*mm*)	
2	Lumen	Minimum lumen area (mm^2^)
3		Mean lumen area (mm^2^)
4		Minimum lumen diameter (mm)
5		Mean lumen diameter (mm)
6	Calcium	Maximum calcium angle (°)
7		Minimum calcium angle (°)
8		Maximum calcium thickness (mm)
9		Minimum calcium thickness (mm)
10		Maximum calcium depth (mm)
11		Minimum calcium depth (mm)
12	FC	Maximum FC angle (°)
13		Minimum FC angle (°)
14		Minimum FC thickness (mm)
15		Maximum FC area (mm^2^)
16		FC surface area (mm^2^)
17		FC burden

### 2.4 Definition of neoatherosclerosis

In this study, neoatherosclerosis was defined as the presence of accumulation of lipid-laden foamy macrophages with or without necrotic core in IVOCT images (17). Two experienced interventional cardiologists manually identified neoatherosclerosis. In case of disagreement between the two readers, they reevaluated the frames and reached a consensus decision.

### 2.5 Statistical analysis

We evaluated the value of atherosclerosis characteristics in pre-stent IVOCT images as predictors of post-stent neoatherosclerosis using various data science approaches. Statistical analyses were performed using R Studio (version 1.4.1717) software. All variables are presented as mean ± standard deviation. A student *t*-test was used for baseline comparisons of clinical and IVOCT plaque characteristics between neoatherosclerosis and no-neoatherosclerosis groups. To assess inter-correlations of IVOCT features, we performed a heatmap analysis using the non-parametric Spearman’s rank correlation coefficient and hierarchical clustering of the individual IVOCT plaque features using the squared Euclidean distance method. A heatmap analysis was very important to eliminate the collinearity of features for further regression. To examine the incremental value of IVOCT plaque features for predicting neoatherosclerosis, univariate and multivariate logistic regression analyses were performed with 95% confidence intervals (CI). Variables that were significant in the univariate analysis (*p* < 0.05) were included in further multivariate analyses. We created receiver operating characteristic (ROC) curves to analyze the ability of pre-stent image features to predict post-stent neoatherosclerosis. Results were evaluated with area under the curve (AUC). The optimal cutoff values for various parameters were determined using the maximum sum of sensitivity and specificity. A *p*-value <0.05 was considered statistically significant.

## 3 Results

### 3.1 Patient samples

This study included 90 patients with multi-vessel disease who had undergone staged PCI with stent implantation. No patients were excluded on the basis of clinical characteristics or image processing results. The median age was 64.0±10.0 years, and 80.0% were men. Among the 90 patients, 55 (61.1%) had hypertension, 57 (63.3%) were current smokers, and 15 (16.7%) had diabetes mellitus. Mean total cholesterol and low-density lipoprotein cholesterol (LDL-C) decreased significantly from baseline to 3 months (*p* < 0.01), and the level of LDL-C was sustained at 18 months. High-density lipoprotein cholesterol did not change over time (*p*>0.05). Details of patient characteristics are provided elsewhere (52).

### 3.2 Baseline comparisons of IVOCT plaque characteristics

Intravascular optical coherence tomography plaque characteristics at baseline were compared for stents that developed neoatherosclerosis (*n* = 19) or did not develop neoatherosclerosis (*n* = 161) at follow-up (Table 2). Longer lesion length was associated with higher prevalence of neoatherosclerosis (35.2 mm vs. 28.1 mm, *p* < 0.05). In the stented segment, the neoatherosclerosis group had smaller mean lumen diameter (2.39 mm vs. 2.68 mm, *p* < 0.05) and larger maximum calcium angle (193.8° vs. 128.1°, *p* < 0.05) than the no-neoatherosclerosis group. Most FC features were significantly associated with neoatherosclerosis. Particularly, the maximum FC area, FC surface area, and FC burden showed the strongest correlations (*p* < 0.00001). Features related to lumen area and calcium depth were not significantly different between the two groups.

**TABLE 2:** Comparison of intravascular optical coherence tomography (IVOCT) plaque features between the neoatherosclerosis (*n* = 19) and no-neoatherosclerosis (*n* = 161) groups Student *t*-test was applied.

Features		Stented segment		
		Neo (*n* = 19)	Non-neo (*n* = 161)	*p*-value
Lesion length (mm)		35.19 ± 8.15	28.12 ± 12.09	<0.05
Lumen	Minimum lumen area (mm^2^)	1.70 ± 0.93	2.45 ± 1.40	0.07
	Mean lumen area (mm^2^)	4.81 ± 1.93	6.02 ± 2.19	0.06
	Minimum lumen diameter (mm)	0.99 ± 0.49	1.13 ± 0.41	0.17
	Mean lumen diameter (mm)	2.39 ± 0.50	2.68 ± 0.48	<0.05
Calcium	Maximum calcium angle (°)	193.75 ± 100.45	128.13 ± 79.90	<0.05
	Minimum calcium angle (°)	18.63 ± 12.37	16.03 ± 9.04	0.22
	Maximum calcium thickness (mm)	1.28 ± 0.19	1.06 ± 0.40	0.07
	Minimum calcium thickness (mm)	0.16 ± 0.12	0.28 ± 0.13	<0.05
	Maximum calcium depth (mm)	0.50 ± 0.13	0.44 ± 0.23	0.25
	Minimum calcium depth (mm)	0.02 ± 0.02	0.04 ± 0.06	0.23
FC	Maximum FC angle (°)	256.63 ± 70.97	135.68 ± 88.10	<0.001
	Minimum FC angle (°)	20.50 ± 4.00	27.69 ± 19.26	0.15
	Minimum FC thickness (mm)	0.01 ± 0.00	0.03 ± 0.03	<0.05
	Maximum FC area (mm^2^)	7.31 ± 1.30	3.64 ± 2.29	<0.00001
	FC surface area (mm^2^)	27.27 ± 7.76	7.53 ± 7.43	<0.00001
	FC burden	2836.81 ± 1967.20	715.92 ± 713.78	<0.00001

### 3.3 Cluster analysis of significant IVOCT plaque features

**FIGURE 3:**
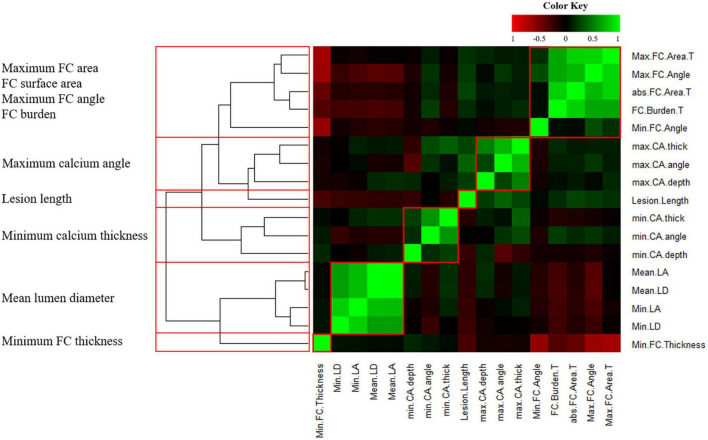
Heatmap of 17 intravascular optical coherence tomography (IVOCT) plaque features with hierarchical clustering. *R*^2^ values are plotted against each other and ordered to show the statistically different clusters (red boxes). The color key indicates the value of explained variances: *R*^2^ values<0.5 are black, whereas greater values are shown in green or red with increasing intensity. Six clusters (red boxes) identified by hierarchical clustering and the IVOCT plaque features with the smallest *p*-values are shown on the left. Using a Spearman correlation coefficient threshold of 0.9, a total of 8 features were excluded from further analysis.

To reduce features that correlated with each other, we performed a correlation heatmap analysis. Figure 3 displays a correlation plot of the 17 IVOCT plaque features, with hierarchical clustering showing distinct clusters of feature variance. With hierarchical clustering, we identified six clusters of IVOCT plaque features that were highly correlated with each other (green areas in the heatmap) but showed minimum relevancy with other features (black areas in the heatmap). Using a Spearman correlation coefficient threshold of 0.9, we eliminated eight IVOCT features (e.g., lumen area, and calcium depth) from further analyses.

### 3.4 Identification of IVOCT plaque features that predict neoatherosclerosis

**FIGURE 4:**
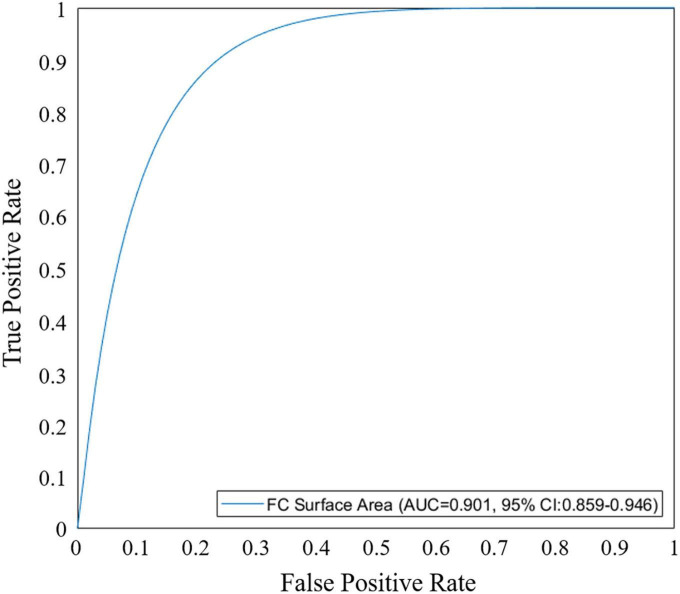
Receiver operating characteristic (ROC) curve analysis of fibrous cap (FC) surface area for predicting in-stent neoatherosclerosis. Area under the curve (AUC) for FC surface area was 0.901 (95% CI 0.859–0.946, *p* < 0.05).

**FIGURE 5:**
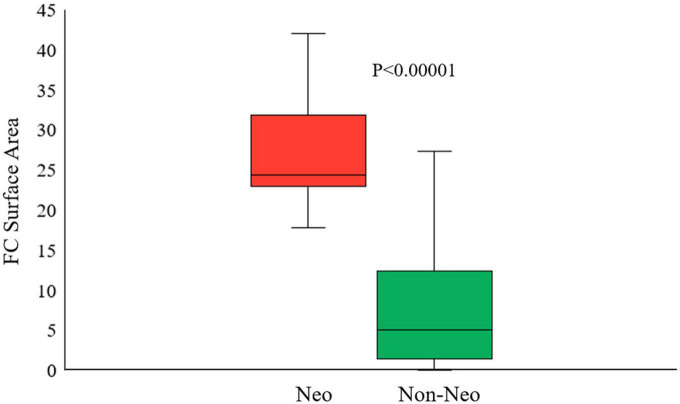
Box plot of the best predictive intravascular optical coherence tomography (IVOCT) plaque feature (FC surface area) showing that the FC surface area was significantly larger in neoatherosclerosis group than that in no-neoatherosclerosis group (*p* < 0.00001). Red indicates neoatherosclerosis and green indicates no-neoatherosclerosis.

**FIGURE 6:**
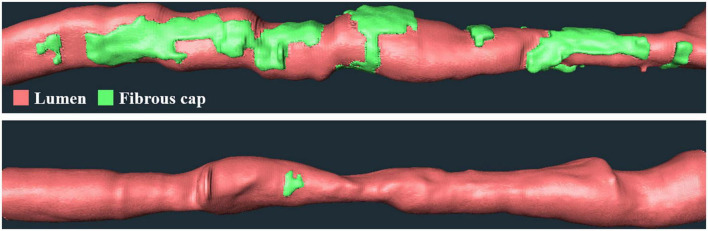
Three-dimensional (3D) visualization of FC surface area on the representative IVOCT pullbacks. Panels are FC distributions of cases (top) with and (bottom) without future neoatherosclerosis. Neoatherosclerosis case had a lesion with a length of 53.4 mm and an FC surface area of 33.1 mm^2^ at baseline, and no-neoatherosclerosis case had a lesion with a length of 37 mm and an FC surface area of 0.73 mm^2^ at baseline. The red is lumen, and the green is FC.

We performed univariate and multivariate logistic regression analyses on the remaining 9 IVOCT plaque features to identify predictors of neoatherosclerosis development. Table 3 lists the IVOCT plaque features associated with neoatherosclerosis by univariate and multivariate logistic regression analyses, including the lesion length and specific lumen features, calcium features, and FC features. In univariate logistic regression analysis, maximum calcium angle, minimum calcium thickness, maximum FC angle, maximum FC area, FC surface area, and FC burden were independent predictors of neoatherosclerosis. However, lesion length and features related to the lumen were not significantly associated. In multivariate logistic regression analysis, only the FC surface area [odds ratio (OR) 1.38, 95% CI 1.05–1.80, *p* < 0.05] was strongly associated with neoatherosclerosis development (Table 3). In a ROC analysis, the AUC was 0.901 (95% CI 0.859–0.946, *p* < 0.05) for FC surface area (Figure 4). In the box plot analysis, FC surface area was significantly larger in the neoatherosclerosis group (*p* < 0.00001, Figure 5). Figure 6 shows 3D visualizations of FC surface area on the representative IVOCT pullbacks (neoatherosclerosis vs. no-neoatherosclerosis).

**TABLE 3:** Univariate/multivariate logistic regression analyses for predicting in-stent neoatherosclerosis.

Features	Univariate			Multivariate		
	OR	95% CI	*p*-value	OR	95% CI	*p*-value
Lesion length (mm)	1.0446	0.99–1.10	0.11			
Maximum calcium angle (°)	1.0084	1.00–1.02	<0.05	1.0084	1.00–1.02	0.21
Minimum calcium thickness (mm)	0.0011	0.00–0.38	<0.05	0.0019	0.00–137.0	0.27
Mean lumen diameter (mm)	0.2324	0.04–1.33	0.10			
Maximum FC angle (°)	1.0183	1.01–1.03	<0.05	0.9992	0.97–1.02	0.95
Minimum FC thickness (mm)	0.0000	0.00–71.65	0.07			
Maximum FC area (mm^2^)	2.2414	1.39–3.61	<0.001	1.3823	0.60–3.17	0.44
FC surface area (mm^2^)	1.3875	1.13–1.71	<0.05	1.3759	1.05–1.80	<0.05
FC burden	1.0016	1.00–1.00	<0.001	0.9998	1.00–1.00	0.82

We found 6 IVOCT plaque features that strongly correlated with neoatherosclerosis formation using univariate logistic regression analysis. In multivariate logistic regression analysis, only the FC surface area was significantly associated with neoatherosclerosis (*p* < 0.05).

## 4 Discussion

To the best of our knowledge, this is the first study to examine pre-PCI IVOCT plaque characteristics as predictors of in-stent neoatherosclerosis. We built on our previous studies (38–49) to evaluate IVOCT-derived plaque features and their association with the development of neoatherosclerosis in follow-up IVOCT examinations. Considering the impact of neoatherosclerosis on patient outcomes, it is important to identify factors that may influence the PCI result. In this study, we automatically extracted multiple IVOCT plaque features prior to PCI, including lesion length, lumen, calcium, and FC features, using a dedicated software (OCTOPUS). Multivariate analysis identified only FC surface area as strongly associated with neoatherosclerosis. In a ROC analysis, the AUC of FC surface area for predicting neoatherosclerosis was 0.901 (95% CI 0.859–0.946, *p* < 0.05).

Optical Coherence TOmography PlaqUe and Stent-enabled automated extraction of IVOCT plaque features was done easily with a reasonable computational time (38). Briefly, the software includes several important functions such as lumen and plaque segmentation, identification of stent struts, and registration of pullbacks for sequential comparisons. The SegNet-based deep learning method (41) was used to segment the lumen boundary in IVOCT images. The calcified plaque was segmented using a two-step deep learning model (46) consisting of preprocessing, determining the major calcification lesions using a 3D convolutional neural network, and segmenting calcified plaques using SegNet. In the context of lipid plaque, we segmented FC by identifying abluminal boundary using dynamic programming (42, 48). Using OCTOPUS, it only took up to 4 min to process a single IVOCT pullback, with manual editing completed within a couple of minutes. Most manual editing was done to optimize the calcium border and lipid arc. The user specifications for OCTOPUS were created in collaboration with interventional cardiologists from the Cardiovascular Imaging Core Laboratory at University Hospitals Cleveland Medical Center, Cleveland, Ohio, a leading laboratory for IVOCT image analysis. OCTOPUS was trained on a large dataset including more than 20,000 clinical images from 150 patients. The software greatly reduces the effort needed to accurately label IVOCT images and is currently used for various offline clinical research purposes by interventional cardiologists. With faster implementation, OCTOPUS-enabled IVOCT data extraction is expected to provide important information for real-time treatment planning.

We observed a strong association between FC surface area and the occurrence of neoatherosclerosis. This finding underscores the importance of automated IVOCT image analysis which can provide quantitative surface area measurements as opposed to a few point estimates of fibrous cap thickness. Although a few studies have investigated the potential contributions of various clinical, stent, and plaque features to the development of neoatherosclerosis, precise understanding of the underlying mechanisms remains unknown. There are multiple potential factors associated with in-stent neoatherosclerosis, such as stent type/age (15, 17, 23, 27–32), clinical factors (24, 27, 33), plaque characteristics (11, 34–36), and stent characteristics (37). Pathological studies first demonstrated a potential association between the prevalence of neoatherosclerosis and stent type and stent age. Nakazawa et al. reported that the prevalence of neoatherosclerosis was significantly greater in lesions with drug-eluting stents than those with bare metal stents, while in-stent neoatherosclerosis occurred more commonly with drug-eluting stents (17). In addition to the stent type and stent age, several clinical characteristics have been related to neoatherosclerosis, such as chronic kidney disease (24, 27), diabetes mellitus (33), low-density lipoprotein cholesterol (27), and lack of treatment with angiotensin-converting enzyme inhibitors or angiotensin II receptor blockers (24). Plaque characteristics have also been implicated in the development of neoatherosclerosis. Studies have suggested that microvessels might be a trigger for neoatherosclerosis development due to a higher prevalence in late in-stent restenotic tissue (11, 35, 36). Neoatherosclerosis has been found to be related to the morphology of the vessel segment (36). Particularly, the presence of lipidous plaque in the stent edges was associated with the occurrence of neoatherosclerosis. Although IVOCT provides an opportunity to conduct comprehensive assessment of coronary plaque, our study is the first to quantitatively analyze plaque characteristics in IVOCT images and their association with outcomes (e.g., neoatherosclerosis).

The findings of the present study have potential clinical relevance. Current interpretation methods for IVOCT images, endorsed by European Society of Cardiology, American Heart Association, and American College of Cardiology, lack assessment of the potential drivers of in-stent neoatherosclerosis. Similar to our findings with IVOCT, intravascular assessment of coronary plaques has been associated with immediate stent deployment outcomes and future events (49, 53). A few studies have investigated the clinical significance of neoatherosclerosis detected by intravascular imaging as a predictor of long-term adverse outcomes in patients undergoing PCI (54, 55). The findings of our study provide insights into the anatomical and inflammatory mechanisms that may link FC surface area with the occurrence of neoatherosclerosis.

This study has some limitations. First, the sample size (*N* = 90) in this serial imaging study was somewhat limited. Therefore, results might be changed with a larger population size. Second, only lipidic neoatherosclerosis was included in this study. Third, it is possible that patient-level factors, such as overall risk of atherosclerosis, might influence the development of neoatherosclerosis after stent placement. Importantly, all patients in this study had multi-vessel disease. Fourth, there may be unmeasured pre-stent plaque characteristics (e.g., microchannel, macrophage infiltration, and cholesterol crystal) that might influence neoatherosclerosis.

The ability to predict the likelihood of developing neoatherosclerosis after PCI can be used by interventional cardiologists to improve treatment planning. Identification of baseline plaque characteristics suggestive of neoatherosclerosis may prompt optimization of treatment to avoid future adverse events such as restenosis (56). Such approaches may inform appropriate planning for optimal stent expansion (20, 57) or the use of new-generation drug-eluting stents (58). Further studies will be necessary to address the clinical utility of IVOCT predictors in improving treatment planning and patient outcomes.

## 5 Conclusion

Neoatherosclerosis can be predicted from pre-stent IVOCT images using quantitative IVOCT plaque characteristics, specifically FC surface area. Our findings highlight the potential clinical benefits of utilizing IVOCT imaging in the catheterization laboratory to inform treatment decision making and optimizing treatment.

## Data availability statement

The original contributions presented in this study are included in the article/supplementary material, further inquiries can be directed to the corresponding author.

## Ethics statement

The studies involving human participants were reviewed and approved by Institutional Review Board of University Hospitals Cleveland Medical Center (Cleveland, OH, USA). The patients/participants provided their written informed consent to participate in this study.

## Author contributions

JL has contributed to the ideation, overview, experiment, and drafting of the manuscript. GP and IM contributed the formal analysis and data preparation. JK contributed to the experiment and drafting of the manuscript. VZ, LD, and SA-K contributed to the data analyses. AH contributed to the ideation. GG contributed to the data curation, review, and editing. DW contributed to the drafting of the manuscript, review, editing, supervision, and funding acquisition. All authors have read and agreed to the submitted version of the manuscript.
